# microRNA-494 Favors HO-1 Expression in Neuroblastoma Cells Exposed to Oxidative Stress in a Bach1-Independent Way

**DOI:** 10.3389/fonc.2018.00199

**Published:** 2018-06-13

**Authors:** Sabrina Piras, Anna L. Furfaro, Rocco Caggiano, Lorenzo Brondolo, Silvano Garibaldi, Caterina Ivaldo, Umberto M. Marinari, Maria A. Pronzato, Raffaella Faraonio, Mariapaola Nitti

**Affiliations:** ^1^Department of Experimental Medicine, University of Genoa, Genoa, Italy; ^2^Department of Molecular Medicine and Medical Biotechnologies, University of Naples “Federico II”, Naples, Italy; ^3^Department of Internal Medicine, Cardiology, Ospedale Policlinico San Martino, University of Genoa, Genoa, Italy; ^4^CEINGE-Biotecnologie Avanzate, Naples, Italy

**Keywords:** heme oxygenase 1, neuroblastoma, miR-494, oxidative stress, Bach1

## Abstract

Heme oxygenase 1 (HO-1) is crucially involved in cell adaptation to oxidative stress and has been demonstrated to play an important role in cancer progression and resistance to therapies. We recently highlighted that undifferentiated neuroblastoma (NB) cells are prone to counteract oxidative stress through the induction of HO-1. Conversely, differentiated NB cells were more sensitive to oxidative stress since HO-1 was scarcely upregulated. In this work, we investigated the role played by miR-494, which has been proved to be involved in cancer biology and in the modulation of oxidative stress, in the upregulation of HO-1. We showed that NB differentiation downregulates miR-494 level. In addition, endogenous miR-494 inhibition in undifferentiated cells impairs HO-1 induction in response to exposure to 500 µM H_2_O_2_, reducing the number of viable cells. The analysis of Bach1 expression did not reveal any significant modifications in any experimental conditions tested, proving that the impairment of HO-1 induction observed in cells treated with miR-494 inhibitor and exposed to H_2_O_2_ is independent from Bach1. Our results underline the role played by miR-494 in favoring HO-1 induction and cell adaptation to oxidative stress and contribute to the discovery of new potential pharmacological targets to improve anticancer therapies.

## Introduction

Heme oxygenase 1 (HO-1) is a 32-kDa inducible enzyme belonging to the HO system, which catalyzes the degradation of the iron-containing molecule heme, leading to the generation of free iron (Fe^2+^), carbon monoxide (CO), and biliverdin. Biliverdin reductase converts biliverdin into bilirubin (1) and ferritin quenches free iron (2). Overall, ferritin, CO, and bilirubin exert strong antioxidant, anti-apoptotic, and anti-inflammatory effects (3). Different activators are involved in HO-1 induction and the nuclear factor erythroid 2-related factor 2 (Nrf2) is considered the most important (4, 5). Moreover, Keap1 by favoring Nrf2 proteasomal degradation, and Bach1 by preventing Nrf2 binding to the promoter region of HO-1, work as HO-1 repressors (6–8).

A sustained HO-1 expression in cancer correlates with a high degree of malignancy (e.g., aggressiveness, metastatic, and angiogenetic potential), although the pro-tumorigenic role of HO-1 seems to be tumor specific and tissue specific (9, 10). In the treatment of highly aggressive neuroblastoma (NB), the upregulation of HO-1 limits the efficacy of bortezomib (11, 12) suggesting HO-1 inhibition may represent a molecular target in the clinical strategies against NB (13, 14).

By modulating the expression of many different proteins, microRNAs (miRs) supervise and integrate numerous signaling pathways and their involvement has been postulated in various physiological and pathophysiological processes, from differentiation to senescence or oncogenesis (15–17). Strong evidence supports the notion that miRs can behave as oncogenes or tumor suppressor genes (18), and given the important role of oxidative stress response in tumorigenesis, understanding miR regulation in this condition is of major interest. Since a role played by miR-494 in the modulation of oxidative stress has been demonstrated in other contexts (19, 20), but no studies have been conducted in NB cells so far. In this work, we aimed at investigating the functional role of miR-494 in NB cell response to oxidative stress, focusing on its involvement in HO-1 induction.

## Materials and Methods

### Cell Culture and Differentiation

SH-SY5Y and SK-N-BE(2C) NB cells were cultured in RPMI 1640 medium (Euroclone, Italy) supplemented with 10% fetal bovine serum (Euroclone), 2 mM glutamine (Sigma-Aldrich, Italy), 1% amphotericin B (Sigma-Aldrich), and 1% penicillin/streptomycin (Sigma-Aldrich). Cells were differentiated by growth in the same medium supplemented with 10 µM all-trans retinoic acid (ATRA) (Sigma-Aldrich) for 4 days, up to 8 days. Differentiation was monitored by checking morphological changes such as neurite elongation and biochemical markers such as MAP2 and NeuroD1 expression (21, 22).

### RNA Extraction and microRNA Level Evaluation

Total RNA was extracted using TRIZOL reagent (Life Technologies, Carlsbad, CA, USA) according to the manufacturer’s instructions. The cDNA templates for evaluation of mature miR levels were obtained from input RNAs (10 ng) using TaqMan™ Advanced miR cDNA Synthesis Kit (Thermo Fisher Scientific, USA, Cat. No. A28007) following the manufacturer’s protocol. Real-time quantitative PCR for hsa-miR-494, hsa-miR-128, hsa-miR-425-5p, and hsa-let7g-5p was performed in triplicate on diluted cDNA templates (1:10) by using the TaqMan^®^ Advanced miR Assays (Thermo Fisher Scientific, Cat. No. A25576). hsa-miR-425-5p and hsa-let7g-5p were used as endogenous reference miRs. Relative quantification of miR expression levels was performed according to the ΔΔCt method.

### Transfection of microRNA Inhibitors

SH-SY5Y cells were transiently transfected with 100 pmol of miR-494 inhibitor (miRCURY LNA miR inhibitor—hsa-miR-494-3p, QIAGEN, Hilder, Germany) and miR inhibitor Control (mirCURY LNA miR inhibitor Control—negative Control A, QIAGEN) by using Lipofectamine 2000 (Life Technologies) following the manufacturer’s instruction. 96 h after transfection, cells were treated with 500 µM H_2_O_2_ for further 6 h to assess Bach1 levels, or for further 24 h to assess HO-1 levels and cell viability.

### Cell Viability Assay

Cell viability was evaluated by Trypan blue assay as previously described (12).

### Reactive Oxygen Species (ROS) Evaluation

Evaluation of ROS was performed by using 2′,7′-dichlorofluorescein diacetate (DCFH-DA; Sigma-Aldrich) assay. After treatments, cells stained with 5 µM DCFH-DA for 30 min at 37°C were analyzed by FACS (Attune™ Acoustic Focusing Flow Cytometer, Thermo Fisher Scientific). Values are expressed as arbitrary units of fluorescence.

### Immunoblotting

Total protein lysates, prepared by using RIPA buffer (13), were subjected to electrophoresis on SDS-polyacrylamide gel (Mini protean precast TGX gel, Bio-Rad, Milan, Italy) (22). Immunodetection was performed using mouse anti-hnRNPQ (1:1,000, Santa Cruz), rabbit anti-PTEN (1:1,000, Cell Signaling Technology, MA, USA), rabbit anti-Bach1 (1:4,000, Bethyl Lab, Montgomery, TX, USA), and rabbit anti-HO-1 (1:2,000, Origene, Herford, Germany) and specific secondary antibodies (GE Healthcare). The membranes were re-probed with the loading control antibodies, rabbit anti-GAPDH (1:1,000, Santa Cruz) or mouse anti-tubulin (1:2,000, AbCam). The bands were detected by means of an enhanced chemiluminescence system (GE Healthcare) and developed films analyzed using a specific software (GelDoc, Bio-Rad).

### Statistical Analyses

Statistical analysis of the differences among mean values ± SEM from three or more experiments was performed by using *t*-test to compare two groups or one-way ANOVA followed by Dunnett’s post-test to compare more groups.

## Results

### ATRA-Induced NB Cell Differentiation Is Associated With Reduced Levels of miR-494

**Figure 1:**
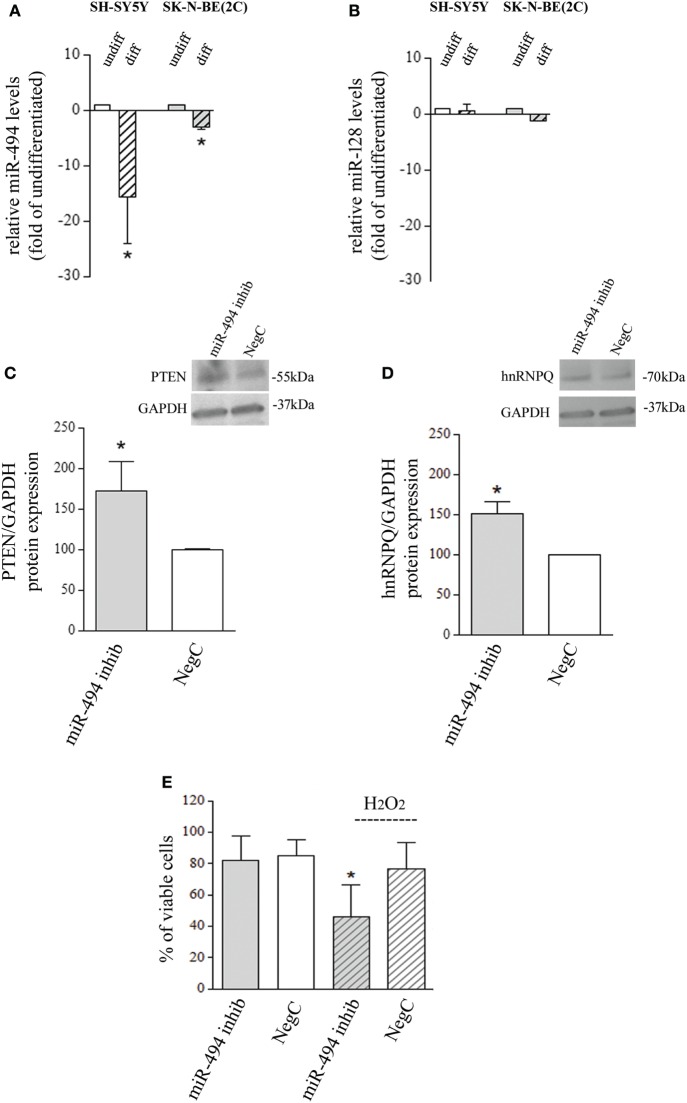
miR-494 downregulation occurs in neuroblastoma (NB) differentiation and modify cell response to H_2_O_2_. **(A)** Expression levels of mature miR-494 in undifferentiated or all-trans retinoic acid (ATRA)-differentiated SH-SY5Y and SK-N-BE(2C) NB cells. hsa-miR-425-5p and hsa-let7g-5p were used as endogenous reference miRs. Results are reported as relative to the values obtained in untreated control cells, which was set equal to 1. Statistical analysis: *n* = 3; *p < 0.05 vs undifferentiated. **(B)** Expression levels of mature miR-128 in undifferentiated or ATRA-differentiated SH-SY5Y and SK-N-BE(2C) NB cells. hsa-miR-425-5p and hsa-let7g-5p were used as endogenous reference miRs. Results are reported as relative to the values obtained in untreated control cells, which was set equal to 1. Statistical analysis: *n* = 3. No significant differences. **(C)** WB analysis of PTEN. GAPDH expression has been used as loading control. 40 µg of protein has been loaded. The bands show the most representative experiment. Statistical analysis: *n* = 3; *p < 0.05 vs NegC. **(D)** WB analysis of hnRNPQ. GAPDH expression has been used as loading control. 40 µg of protein has been loaded. The bands show the most representative experiment. Statistical analysis: *n* = 2; *p < 0.05 vs NegC. **(E)** Percentage of viable cells (Trypan blue analysis) after miR-494 inhibition and 24 h exposure to 500 µM H_2_O_2_. Statistical analysis: *n* = 4; *p < 0.05 vs NegC and miRNA 494 inhibitor.

The analysis of miR-494 expression, performed after 4-day exposure to 10 µM ATRA, showed a significant reduction in both SH-SY5Y and SK-N-BE(2C) cell lines (Figure 1A). SH-SY5Y cells increase the expression of differentiation markers already after 4-day exposure to ATRA, as widely proved (21–23). However, since SK-N-BE(2C) need more time to complete ATRA-induced differentiation (24), miR evaluation was also performed after 6- and 8-day exposure to 10 µM ATRA on SK-N-BE(2C) cells. In these conditions, only a small further decrease of miR-494 expression has been observed (Figure S1a in Supplementary Material). The expression of miR-128 has been also analyzed due to its involvement in stress response and differentiation (25, 26), but no changes were observed in both cell lines after differentiation (Figure 1B). The following experiments have been carried out on SH-SY5Y NB cells which strongly downregulated miR-494 (−10-folds vs undifferentiated) in the shortest experimental time (4 days).

### miR-494 Inhibition Modifies Cell Responses to Oxidative Stress

To evaluate whether the reduction of endogenous miR-494 could modify NB cell sensitivity to oxidative stress, cells were transfected with a specific miR-494 inhibitor and then exposed to 500 µM H_2_O_2_. The effectiveness of miR-494 inhibition was checked by evaluating the protein levels of two miR-494 targets, namely, PTEN and hnRNPQ that resulted upregulated of about 50% (Figures 1C,D). The analysis of viable cells revealed no changes induced by miR-494 inhibition itself, in comparison to cells transfected with a NegC. Conversely, miR-494 inhibition significantly decreased the percentage of viable cells after the exposure to 500 µM H_2_O_2_ (Figure 1E). The analysis of markers of apoptosis such as BAX and PARP did not show any changes (Figures S1b and S1c in Supplementary Material) and this led us to rule out the occurrence of apoptosis. These results indicate that the expression of miR-494 in undifferentiated NB cells favors cell adaptation/response to oxidative stress.

### miR-494 Inhibition Impairs HO-1 Induction in Response to Oxidative Stress

**Figure 2:**
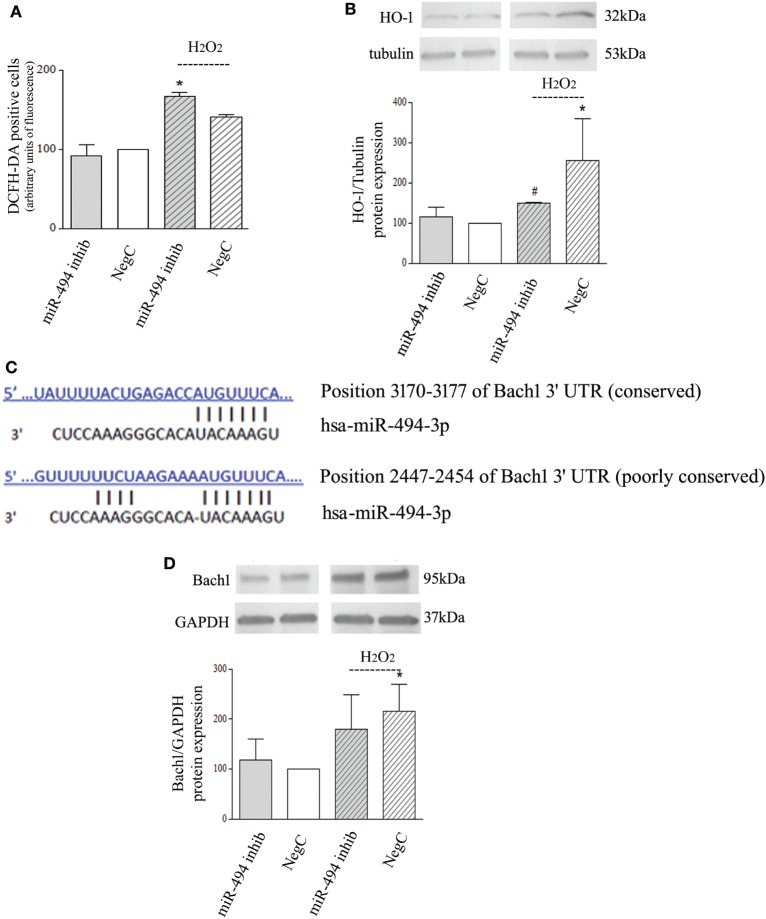
miR-494 inhibition impairs heme oxygenase 1 (HO-1) induction in response to H_2_O_2_ in Bach1-independent way. **(A)** Positivity to DCFH-DA has been measured by cytofluorimetric analyses after miR-494 inhibition and 6 h exposure to 500 µM H_2_O_2_. Statistical analysis: *n* = 2; *p < 0.05 vs NegC. **(B)** WB analysis of HO-1 expression. Tubulin expression has been used as loading control. 30 µg of protein has been loaded. The bands show the most representative experiment. Statistical analysis: *n* = 3; *p < 0.05 vs NegC. ^#^*p* < 0.05 vs NegC + H_2_O_2_. **(C)** The human Bach1 3′UTR contains two seed sites for miR-494. The sequence alignments were predicted using TargetScan. **(D)** WB analysis of Bach1 expression. GAPDH expression has been used as loading control. 30 µg of protein has been loaded. The bands show the most representative experiment. Statistical analysis: *n* = 4; *p < 0.05 vs NegC.

To investigate whether a reduced expression of miR-494 influences oxidative stress response, NB cells transfected with miR-494 inhibitor or NegC were treated with H_2_O_2_ and ROS levels and HO-1 expression were evaluated. ROS levels were increased only in cells treated with miR-494 inhibitor and exposed to H_2_O_2_ (Figure 2A). In this experimental condition, no significant induction of HO-1 has been observed (Figure 2B). Conversely, in NB cells transfected with NegC, the exposure to H_2_O_2_ was able to significantly increase the expression of HO-1, and the level of ROS was not significantly modified.

The ubiquitination pattern was also analyzed but no changes were detected in any experimental conditions (Figure S1d in Supplementary Material).

*In silico* analyses predicted Bach1 as a target of miR-494 with two putative sites within its 3′UTR (Figure 2C). Thus, we checked the protein levels of Bach1. WB analysis showed that miR-494 inhibition did not modify Bach1 expression after H_2_O_2_ exposure (Figure 2D). Different Bach1 post-translational modifications have also been analyzed; ubiquitination and sumoylation were not detected and Bach1 acetylation was not modified in any experimental conditions (Figure S1e in Supplementary Material). These results show no involvement of Bach1 in the miR-494 dependent HO-1 regulation.

Furthermore, Keap1 levels have been also checked but no changes were detected in any experimental conditions (Figure S1f in Supplementary Material), proving no involvement of Nrf2 in this context.

## Discussion

In this work, we pointed out the involvement of miR-494 in the upregulation of HO-1 in NB cell response to oxidative stress. We took into consideration two miRs, such as miR-128 and miR-494. Indeed, miR-128 has been demonstrated to be involved in NB differentiation (26) and response to oxidative stress (25) but we did not observe any modification of miR-128 levels in the different experimental conditions we tested. Thus, we evaluated miR-494 which, from bioinformatics analyses, was predicted to have two putative binding sites on Bach1 3′UTR, the main repressor of HO-1 transcription.

In numerous contexts, miR-494 functions as tumor suppressor gene and has been linked to the induction of senescent phenotype in normal cells (19, 27) but in other contexts it correlates with tumor aggressiveness and progression (28). To the best of our knowledge, there has been no evidence of miR-494 expression in NB so far. We demonstrated that miR-494 is expressed in two undifferentiated NB cell lines and undergoes a significant downregulation after ATRA-induced differentiation. The reduction is dramatic for SH-SY5Y cells that easily differentiate in response to ATRA and minor but always significant in SK-N-BE(2C) which shown medium sensitivity to ATRA (24). There is only a paper in literature showing that the expression of miR-494 is upregulated by ATRA in the acute myeloid leukemia cell line HL-60 (29), and this lets us hypothesize that there may be a cell-type-specific regulation for miR-494. Thus, we further analyzed SH-SY5Y cells which, from our previous works, have been proved to increase their sensitivity to oxidative stress after differentiation (22, 30), investigating a possible correlation with the miR-494 downregulation. We observed that miR-494 inhibition in undifferentiated cells significantly reduced the number of viable cells after exposure to H_2_O_2_. The role of miR-494 in cell survival is controversial, depending on the cellular context where miR operates and on the accessibility of its targets. As also shown in our work, miR-494 inhibition is able to increase PTEN expression and, potentially, to antagonize the AKT survival pathway, as proved in other contexts (31, 32). However, the modulation of miR-494-PTEN signaling under stress condition has not yet been investigated.

Next, we provided evidence that endogenous miR-494 inhibition impairs HO-1 upregulation in response to oxidative stress, similar to what we have already shown in differentiated cells exposed to H_2_O_2_ (22). In addition, we showed that the lack of HO-1 upregulation correlates with higher ROS levels, highlighting the importance of HO-1 induction in quenching ROS. However, the analysis of Bach1 expression revealed that there are no significant modifications in the level of Bach1 in response to H_2_O_2_ in NB cells treated with miR-494 inhibitor compared with cells transfected with NegC. Moreover, there are no changes in Bach1 ubiquitination, sumoylation, and acetylation in any experimental conditions examined. Thus, miR-494 could contribute through a Bach1-independent mechanism to modulate HO-1 expression under stress response. It has been already demonstrated that HO-1 transcription can be controlled *via* Bach1 turnover in the absence or presence of oxidative stress and can also be insensitive to Bach1-mediated repression (33). Moreover, AKT-dependent HO-1 induction has been already proved (34), and miR-494-PTEN might crucially modulate it. To validate this hypothesis, further analyses are needed.

## Author Contributions

SP, ALF, SG, RF, and MN conceived and designed the experiments. SP, ALF, RC, LB, SG, and CI conducted the experiments. SP, ALF, SG, RF, and MN analyzed the results. UMM, MAP, RF, and MN contributed reagents/materials/analysis. SP, ALF, UMM, RF, and MN wrote the paper. All the authors reviewed the manuscript.

## Conflict of Interest Statement

The authors declare that the research was conducted in the absence of any commercial or financial relationships that could be construed as a potential conflict of interest.

## Supplementary Material

FIGURE S1 **(A)** Expression levels of mature miR-494 and miR-128 in undifferentiated and 6- or 8-day-differentiated SK-N-BE(2C) NB cells. hsa-miR-425-5p and hsa-let7g-5p have been used as endogenous reference miRs. Results are reported as relative to the values obtained in untreated undifferentiated cells which was set equal to 1. Statistical analysis: n = 3; **p* < 0.05 vs undifferentiated. **(B)** WB analysis of BAX in SH-SY5Y cells treated with miR-494 inhibitor and exposed to 500mM H_2_O_2_, as indicated. GAPDH expression has been used as loading control. 10 mg of proteins was loaded. The blots show one representative experiment. Statistical analysis: n = 3; no significant differences. **(C)** WB analysis of PARP in SH-SY5Y cells treated with miR-494 inhibitor and exposed to 500mM H_2_O_2_, as indicated. GAPDH expression has been used as loading control. 50 mg of proteins was loaded. The blots show one representative experiment. Statistical analysis: n = 2; no significant differences. **(D)** WB analysis of ubiquitination in SH-SY5Y cells treated with miR-494 inhibitor and exposed to 500mM H_2_O_2_, as indicated. 20 mg of proteins was loaded. The blot shows one representative experiment. **(E)** Analysis of Bach1 post-translational modifications in SH-SY5Y cells treated with miR-494 inhibitor and exposed to 500mM H_2_O_2_ for 6 h. 300 mg of protein lysate was immunoprecipitated using anti Bach1 and loaded in electrophoresis (ip). An aliquot of supernatant collected after the first step of immunoprecipitation was loaded in electrophoresis (sn). WB detection was performed as indicated. The blots show the most representative experiment. **(F)** WB analysis of Keap1 in SH-SY5Y cells treated with miR-494 inhibitor and exposed to 500mM H_2_O_2_ as indicated. GAPDH expression has been used as loading control. 40 mg of proteins was loaded. The blots show one representative experiment. Statistical analysis: n = 3; no significant differences.
