# Supplementary figures and images for: microRNA-494 Favors HO-1 Expression in Neuroblastoma Cells Exposed to Oxidative Stress in a Bach1-Independent Way
Source: Front Oncol. 2018 Jun 13;8:199. doi: 10.3389/fonc.2018.00199 (PMC6008388; doi:10.3389/fonc.2018.00199)

**a**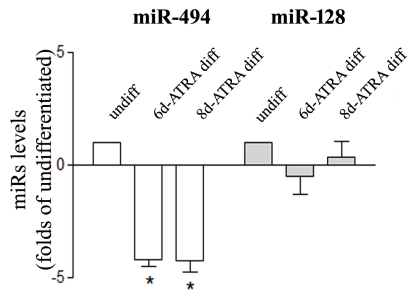**b**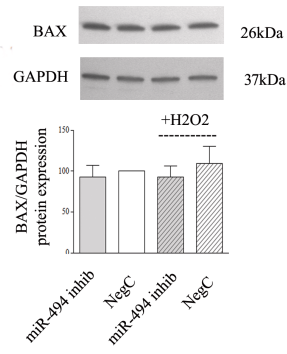**c**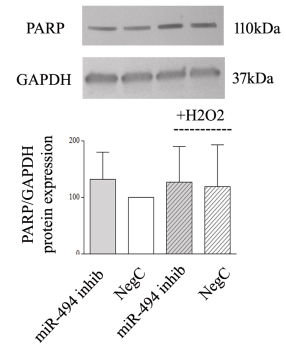**d**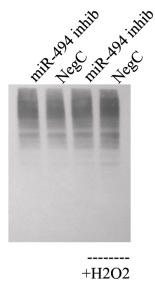**e**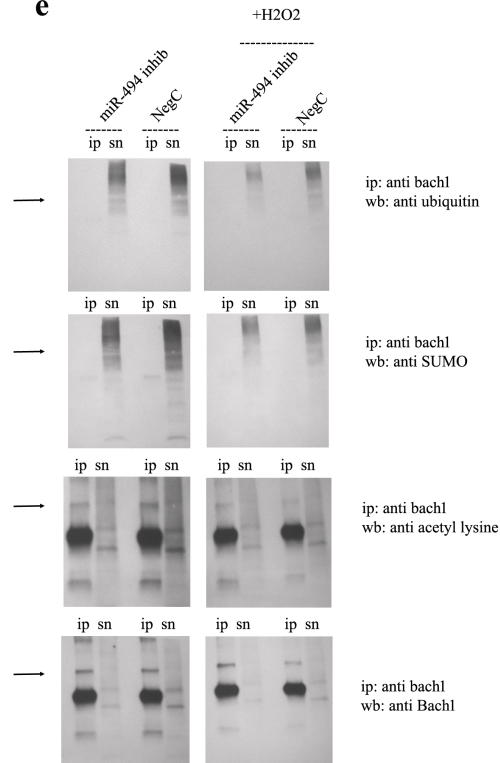**f**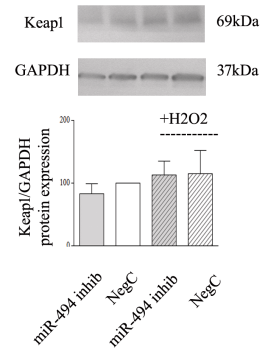

Supplement: FIGURE S1 — (A) Expression levels of mature miR-494 and miR-128 in undifferentiated and 6- or 8-day-differentiated SK-N-BE(2C) NB cells. hsa-miR-425-5p and hsa-let7g-5p have been used as endogenous reference miRs. Results are reported as relative to the values obtained in untreated undifferentiated cells which was set equal to 1. Statistical analysis: n = 3; *p < 0.05 vs undifferentiated. (B) WB analysis of BAX in SH-SY5Y cells treated with miR-494 inhibitor and exposed to 500mM H2O2, as indicated. GAPDH expression has been used as loading control. 10 mg of proteins was loaded. The blots show one representative experiment. Statistical analysis: n = 3; no significant differences. (C) WB analysis of PARP in SH-SY5Y cells treated with miR-494 inhibitor and exposed to 500mM H2O2, as indicated. GAPDH expression has been used as loading control. 50 mg of proteins was loaded. The blots show one representative experiment. Statistical analysis: n = 2; no significant differences. (D) WB analysis of ubiquitination in SH-SY5Y cells treated with miR-494 inhibitor and exposed to 500mM H2O2, as indicated. 20 mg of proteins was loaded. The blot shows one representative experiment. (E) Analysis of Bach1 post-translational modifications in SH-SY5Y cells treated with miR-494 inhibitor and exposed to 500mM H2O2 for 6 h. 300 mg of protein lysate was immunoprecipitated using anti Bach1 and loaded in electrophoresis (ip). An aliquot of supernatant collected after the first step of immunoprecipitation was loaded in electrophoresis (sn). WB detection was performed as indicated. The blots show the most representative experiment. (F) WB analysis of Keap1 in SH-SY5Y cells treated with miR-494 inhibitor and exposed to 500mM H2O2 as indicated. GAPDH expression has been used as loading control. 40 mg of proteins was loaded. The blots show one representative experiment. Statistical analysis: n = 3; no significant differences. [file image_1.PDF]
