# Supplementary material for: microRNA-494 Favors HO-1 Expression in Neuroblastoma Cells Exposed to Oxidative Stress in a Bach1-Independent Way
Source: Front Oncol. 2018 Jun 13;8:199. doi: 10.3389/fonc.2018.00199 (PMC6008388; doi:10.3389/fonc.2018.00199)
Supplement: Supplementary file 2 [file data_sheet_1.docx]

**Supplementary Materials and Methods**

**Immunoblotting**

Using the same method described in the manuscript, the following antibodies have been used

-rabbit anti BAX (1:500, AbCam, UK)

-rabbit anti PARP (1:1000, Cell signaling Technology, USA)

-mouse anti ubiquitin (1:1000, Santa Cruz Biotech, USA)

**Immunoprecipitation**

Starting from 300μg of total lysate, Bach1 has been immunoprecipitated using 1μg of rabbit anti Bach1 (Bethyl Lab, USA) incubated ON at 4°C and 30 μl of Dynabeads prot.G (Novex, Life Technologies) incubated 1h at 4°C, following the manufacturer protocol.

Samples from IP have been subjected to electrophoresis as described for WB and the following antibodies have been used to detect Bach1 modifications:

-mouse anti ubiquitin (1:1000, Santa Cruz Biotech, USA)

-mouse anti SUMO2/3 (1:5000, AbCam, UK)

-rabbit anti acetyl lysine (1:2000, Cell Signaling)

**Supplementary figure 1 legend**

a) Expression levels of mature miR-494 and miR-128 in undifferentiated and 6day- or 8day-differentiated SK-N-BE(2C) NB cells. hsa-miR-425-5p and hsa-let7g-5p have been used as endogenous reference miRs. Results are reported as relative to the values obtained in untreated undifferentiated cells which was set equal to 1. Statistical analysis: n=3; *p<0.05 vs undifferentiated b) WB analysis of BAX in SH-SY5Y cells treated with miR-494 inhibitor and exposed to 500μM H_2_O_2_, as indicated. GAPDH expression has been used as loading control. 10μg of proteins were loaded. The blots show one representative experiment. Statistical analysis: n=3; no significant differences. c) WB analysis of PARP in SH-SY5Y cells treated with miR-494 inhibitor and exposed to 500μM H_2_O_2_, as indicated. GAPDH expression has been used as loading control. 50μg of proteins were loaded. The blots show one representative experiment. Statistical analysis: n=2; no significant differences. d) WB analysis of ubiquitination in SH-SY5Y cells treated with miR-494 inhibitor and exposed to 500μM H_2_O_2_, as indicated. 20 μg of proteins were loaded. The blot shows one representative experiment. e) Analysis of Bach1 post-translational modifications in SH-SY5Y cells treated with miR-494 inhibitor and exposed to 500μM H_2_O_2_ for 6h. 300μg of protein lysate were immunoprecipitated using anti Bach1 and loaded in electrophoresis (ip). An aliquot of supernatant collected after the first step of immunoprecipitation was loaded in electrophoresis (sn). WB detection was performed as indicated. The blots show the most representative experiment.
f) WB analysis of Keap1 in SH-SY5Y cells treated with miR-494 inhibitor and exposed to 500μM H_2_O_2_ as indicated. GAPDH expression has been used as loading control. 40μg of proteins were loaded. The blots show one representative experiment. Statistical analysis: n=3; no significant differences.
